# The Effect of Satellite Laboratory Installation on Chemotherapy Waiting Time in an Oncology Treatment Unit

**DOI:** 10.7759/cureus.2333

**Published:** 2018-03-16

**Authors:** Abdullah Bany Hamdan, Abdullah Aldakhil, Mushabbab Asiri, Jesusa Tamani, Sherwynn Javison, Sheena Peethambaran, Isamme AlFayyad, Richard Erlandez, Huda AlKarim, Musa AlHarbi

**Affiliations:** 1 Comprehensive Cancer Center, King Fahad Medical City, Riyadh, Saudi Arabia; 2 Management Department, College of Business Administration, King Saud University, Riyadh, Saudi Arabia; 3 Research Center, King Fahad Medical City, Riyadh, Saudi Arabia

**Keywords:** satellite laboratory, chemotherapy, oncology, waiting time, cancer

## Abstract

Background

Timely treatment is a patient's right. Increasing the efficiency of laboratory testing could potentially improve hospital operations, provide quicker access to health services, and have a positive impact on patient experience. Installation of a satellite laboratory may shorten laboratory turnaround time (TAT) and chemotherapy waiting time.

Method

The laboratory TAT and chemotherapy waiting time were analyzed and compared before and three years after the establishment of the satellite laboratory.

Result

The mean laboratory TAT decreased from one hour and 30 minutes at baseline in 2012 to 43, 43, and 37 minutes in 2013, 2014, and 2015, respectively; a reduction rate of 59%. Mean chemotherapy waiting time also reduced, from a 2012 baseline of 252 minutes to 170, 157, and 146 minutes in 2013, 2014, and 2015, respectively; a reduction rate of 42%.

Conclusion

The reduced chemotherapy waiting time after the installation of a satellite laboratory had a positive impact on patient care. It also reduced employee workload and maximized the utilization of hospital resources.

## Introduction

One of the more important goals in meeting quality care standards is maximizing patient experience concerning access to health services. Providing care for a cancer patient is challenging due to the complexity of the available therapeutic modalities, especially chemotherapy. In this regard, it is essential that patients receive their course of treatment on time. However, some hospitals providing cancer care and treatment face challenges in administering chemotherapy in a safe, efficient, and timely manner because of the increasing number of patients who require chemotherapy [1]. Methods of determining appropriate treatments specific to patient diagnosis are now being studied and implemented [1].

Chemotherapy waiting time is defined as the time from patient check-in to the administration of treatment [2]. Chemotherapy waiting time is an important indicator of cancer center efficiency in providing chemotherapy on an outpatient basis. Selecting the appropriate treatment involves a complex process that includes making an appointment, followed by physician examination, laboratory testing, and the prescription and preparation of medication followed by the actual administration of the chemotherapeutic agent(s). All these steps require time and involve encounters with several hospital employees [2]. However, before treatment is administered to a patient, it is important that all required laboratory tests and other important examinations be performed. After receiving the results of these tests, the physician will determine the specific treatment appropriate for the condition of the patient. Delays in care and the length of time that patients wait in the hospital for treatment to begin can have a major impact on patient and staff satisfaction [3].

Increasing the efficiency of laboratory testing could potentially improve hospital operations and thus have a positive impact on patient care [4]. Testing efficiency may be enhanced by installing a satellite laboratory in a specific department in a hospital. Satellite laboratories are usually small branch laboratories that are affiliated with but not physically located at the central laboratory. In most cases, the satellite laboratory services are limited. However, studies revealed that “the satellite laboratory now provides a useful service that has improved both the timeliness and quality of patient care” [5]. Laboratory results are needed for the physician to make a sound clinical decision, and this is one of the most frequent causes of delayed treatment [6]. This is especially applicable to patients receiving chemotherapy, who wait for an extended period for laboratory results prior to starting chemotherapy [7]. Lengthening the time required to perform a laboratory procedure and process its results lengthens the time before the physician can prescribe the chemotherapeutic agent. Both patients and healthcare providers are frustrated at these delays. Moreover, increases in the numbers of patients having to undergo chemotherapy sessions have increased the frustration levels of both patients and healthcare providers. Some oncologists have therefore considered the establishment of satellite laboratories to serve the diagnostic needs of patients receiving chemotherapy [7]. Although delays in care may be inevitable, they may also result from poor management and result in unplanned and irrational scheduling and resource allocation [3]. By monitoring chemotherapy waiting time, an institution can allocate existing resources and plan for more efficient and timely service.

Improving access to diagnostic procedures is an essential component of health service strategies to improve access to care. Turnaround time (TAT) is regarded by most clinicians as a factor associated with the quality of laboratory services. TAT is operationally defined as the amount of time taken to fulfill a request, from the time of receipt at the laboratory to the verification of results. Studies have shown that compared with central laboratory testing, satellite laboratory testing may significantly reduce the waiting times for both clinicians and patients, thus providing a smoother flow of health services to patients receiving treatment [4]. TAT is a measurable parameter of laboratory services, as well as being a key indicator of laboratory performance [8]. The primary benefit of satellite laboratories is to improve patient care. Thus, reducing TAT should reduce total patient costs for immediate and/or long-term care [9].

This problem has been encountered by the oncology outpatient treatment unit of the Comprehensive Cancer Center of King Fahad Medical City in Riyadh, Saudi Arabia. Staff nurses obtain blood samples and send them to the central laboratory. This process is regarded as an addition to the nursing workload of caring for multiple patients. This has lengthened the time required to obtain laboratory test results needed to confirm whether a patient is fit to receive a chemotherapy agent. Thus, this process delays the preparation of the chemotherapy agent in the pharmacy, as well as lengthens the time prior to the administration of drug to the patient. Assessments of patient feedback have confirmed disappointment because of extended chemotherapy waiting times, with patients believing that their holistic needs have not been met. Installation of a satellite laboratory may improve the care of patients receiving chemotherapy.

Improvement in the quality of patient care is an essential aspect of caring. High-performing health care systems have been described as those that are patient-centric, equitable, efficient, accessible, and recognize quality as a systems property. Nurses can bring specimens directly and quickly to a satellite laboratory. Results can be obtained as soon as possible, thereby enhancing integrated patient care [4].

Quick access to treatment, when needed, is a hallmark of a high-quality healthcare system. However, this is a critical problem in most countries, and restructuring care delivery to cancer patients is essential to quality improvement. Waiting time is the main component of patient care that can be improved. Longer wait times have been associated with reduced patient satisfaction [10]. Patient-centered care means identifying the need for a specific patient population, which includes redesigning internal processes, if needed, to improve patient satisfaction and outcome [11]. Patient satisfaction is one of several factors that must be considered in determining the quality of care. Furthermore, the overall quality of service is determined by the system and not by the individual [12]. Advanced access to care is based on five principles: understanding demand, shaping the handling of demand by providing alternatives to face-to-face consultations, matching capacity to demand, developing contingency plans, and communicating continuously with staff and patients [13].

The main purpose of this study was to determine the advantages of having a satellite laboratory in an oncology outpatient treatment unit. Moreover, this study assessed whether the presence of a satellite laboratory could help reduce laboratory TAT and chemotherapy waiting time for cancer patients.

## Materials and methods

Research locale

The study was performed in the adult oncology treatment unit at the Comprehensive Cancer Center of King Fahad Medical City in Riyadh, Saudi Arabia. The satellite laboratory had been installed in this unit.

Research design

Laboratory TAT and chemotherapy waiting time were collected retrospectively prior to the installation of the satellite laboratory in 2012. Similar data were collected yearly from 2013 to 2015, after installation of the satellite laboratory.

Population

The respondents include cancer patients who received ongoing chemotherapy treatment, regardless of age, diagnosis, or gender. Patients who came in a day before their appointment and provided early specimens for collection were excluded. Similarly, patients who came to the outpatient unit for procedures only, and those with deferred appointments, were excluded.

Sampling technique

A simple random sampling technique was used to choose samples and generate data by tracking TAT from obtaining laboratory samples to the release of test results, and from the start to the end of chemotherapy sessions.

Research instrument

The English language version of the study tracking tool was used. The accuracy of this instrument was assessed and validated by a series of reviews, verifications, and pilot tests performed by an expert panel. The tool included the time each specimen was sent to the laboratory, the time that the results of blood tests were available, the time the patient arrived at the unit, the time the chemotherapy order was sent to the pharmacy, the time required to dispense the chemotherapeutic agent, the time required for pre-medication, the required for chemotherapy administration, and the time for completion of chemotherapy.

Measures and analyses

Chemotherapy waiting times before and after the installation of the satellite laboratory were reported as mean ± standard deviation (SD) and compared by paired sample t-tests. A p-value <0.05 was considered statistically significant. The degree of association between the laboratory TAT and chemotherapy waiting time before interventions were analyzed by calculating Pearson’s r correlation coefficient. All statistical analyses were performed using IBM Statistical Package for the Social Sciences (SPSS) for Windows, version 22.0 (IBM Corp., Armonk, NY).

Ethical considerations

The research was approved by the Institutional Review Board (IRB) of our hospital. None of the authors reported any conflicts of interest.

## Results

Prior to the installation of the satellite laboratory, there was a significant relationship (p = 0.009) between the laboratory TAT and the chemotherapy waiting time, as well as a moderate correlation (r = 0.365) between the two variables (Table 1).

**Table 1:** Correlation between laboratory turnaround time and chemotherapy waiting time at baseline in 2012 SD: standard deviation.

	Minimum (min)	Maximum (max)	Mean ± SD (min)	Correlation (r)	P-value
Pre-intervention laboratory turnaround time	31	240	90 ± 42	0.365	*0.009
Pre-intervention chemotherapy waiting time	65	460	252 ± 77		

A comparison of laboratory TAT before 2012 and after the 2013-2015 installation of the satellite laboratory showed that the mean ± SD laboratory TAT decreased significantly, from 90 ± 42 minutes in 2012 to 37 ± 14 minutes in 2015 (p <0.001), a 59% reduction rate three years after the satellite laboratory was established (Table 2).

**Table 2:** Laboratory turnaround times before and after the establishment of a satellite laboratory *Relative to pre-intervention chemotherapy laboratory TAT, 2012. SD: standard deviation; TAT: turnaround time.

	N	Mean ± SD, min	P-value*
Pre-intervention laboratory TAT, 2012	50	90 ± 42	
Post-intervention laboratory TAT, 2013	50	43 ± 22	< 0.001
Post-intervention laboratory TAT, 2014	50	43 ± 14	< 0.001
Post-intervention laboratory TAT, 2015	50	37 ± 14	< 0.001

Chemotherapy waiting time was also significantly reduced after the laboratory TAT was efficiently managed. Mean chemotherapy waiting time improved progressively after the satellite laboratory was installed. The mean ± SD average waiting time at baseline in 2012 was 252 ± 77 minutes, it decreased to 146 ± 31 minutes in 2015 (p <0.001); a 42% reduction rate three years after the satellite laboratory was established (Table 3).

**Table 3:** Average wait time to start chemotherapy from the first blood test appointment *Relative to pre-intervention chemotherapy waiting time, 2012 SD: standard deviation.

	N	Mean ± SD, min	P-value*
Pre-intervention chemotherapy waiting time, 2012	150	252 ± 77	
Post-intervention chemotherapy waiting time, 2013	150	170 ± 52	< 0.001
Post-intervention chemotherapy waiting time, 2014	150	157 ± 34	< 0.001
Post-intervention chemotherapy waiting time 2015	150	146 ± 31	< 0.001

## Discussion

Creating a satellite laboratory may be a costly strategy, but it can have a high impact and enhance care delivery systems. The results of this study showed that the establishment of the satellite laboratory in the oncology treatment unit was beneficial and necessary as a strategy for reducing patient waiting times. Improving the timely delivery of service enhances the overall patient experience through effective coordination and process change [14].

An earlier release of laboratory results correlated with a shorter waiting time for chemotherapy administration. Both the patients and the staff provided positive feedback with regards to having a satellite laboratory. The presence of the satellite laboratory helped reduce staff workload and relieved staff frustration by shortening the time nurses spent transporting specimens and waiting for test results. Moreover, staff members were able to see more patients after the satellite laboratory installation.

Patients deserve to have access to high-quality care, and this is affected by the timeliness and effectiveness of available services [2]. A successful outcome depends on the unified perception of the stakeholders and multidisciplinary participation, with regular process evaluation and transparency of shared results [14]. This study helped in improving patient experience by enhancing prompt and efficient care.

## Conclusions

Installation of a satellite laboratory significantly reduced the mean chemotherapy waiting time by about 50%. This intervention had a positive impact on the patient care process by reducing the length of waiting times from laboratory encounters to chemotherapy administrations. Staff members were less frustrated with their workloads, providing opportunities for the maximum utilization of hospital resources. After the end of the three-year study in 2015, the two administrators of our unit decided that the satellite laboratory in the oncology treatment unit should be permanent, as well as fully operational during business hours. Simultaneously, process guidelines and algorithms were modified to make the care delivery service efficient and maintain the sustainability of improvement.
